# Coccidian Parasites and Conservation Implications for the Endangered Whooping Crane (*Grus americana*)

**DOI:** 10.1371/journal.pone.0127679

**Published:** 2015-06-10

**Authors:** Miranda R. Bertram, Gabriel L. Hamer, Karen F. Snowden, Barry K. Hartup, Sarah A. Hamer

**Affiliations:** 1 Department of Veterinary Integrative Biosciences, Texas A&M University, College Station, Texas, United States of America; 2 Department of Entomology, Texas A&M University, College Station, Texas, United States of America; 3 Department of Veterinary Pathobiology, Texas A&M University, College Station, Texas, United States of America; 4 International Crane Foundation, Baraboo, Wisconsin, United States of America; 5 Department of Surgical Sciences, University of Wisconsin, Madison, Wisconsin, United States of America; University of Florida, UNITED STATES

## Abstract

While the population of endangered whooping cranes (*Grus americana*) has grown from 15 individuals in 1941 to an estimated 304 birds today, the population growth is not sufficient to support a down-listing of the species to threatened status. The degree to which disease may be limiting the population growth of whooping cranes is unknown. One disease of potential concern is caused by two crane-associated *Eimeria* species: *Eimeria gruis* and *E*. *reichenowi*. Unlike most species of *Eimeria*, which are localized to the intestinal tract, these crane-associated species may multiply systemically and cause a potentially fatal disease. Using a non-invasive sampling approach, we assessed the prevalence and phenology of *Eimeria* oocysts in whooping crane fecal samples collected across two winter seasons (November 2012–April 2014) at the Aransas National Wildlife Refuge along the Texas Gulf coast. We also compared the ability of microscopy and PCR to detect *Eimeria* in fecal samples. Across both years, 26.5% (n = 328) of fecal samples were positive for *Eimeria* based on microscopy. Although the sensitivity of PCR for detecting *Eimeria* infections seemed to be less than that of microscopy in the first year of the study (8.9% vs. 29.3%, respectively), an improved DNA extraction protocol resulted in increased sensitivity of PCR relative to microscopy in the second year of the study (27.6% and 20.8%, respectively). The proportion of positive samples did not vary significantly between years or among sampling sites. The proportion of *Eimeria* positive fecal samples varied with date of collection, but there was no consistent pattern of parasite shedding between the two years. We demonstrate that non-invasive fecal collections combined with PCR and DNA sequencing techniques provides a useful tool for monitoring *Eimeria* infection in cranes. Understanding the epidemiology of coccidiosis is important for management efforts to increase population growth of the endangered whooping crane.

## Introduction

The whooping crane (*Grus americana*) experienced a severe population decline in the first part of the 20^th^ century and has been listed as endangered since 1967. The species has rebounded from a low of 15 individuals in 1941 to a total of 451 wild birds, including reintroduced populations, and 157 captive birds in 2013 [1]. The Aransas-Wood Buffalo population (AWBP), which nests in Wood Buffalo National Park, Alberta and Northwest Territories, Canada and winters among coastal marshes in and around the Aransas National Wildlife Refuge in Texas, USA, is the only self-sustaining wild population of whooping cranes. During the 2013–2014 winter, the population was estimated at 304 individuals (95% CI = 260–354; CV = 0.08) [2]. The International Recovery Plan [3] sets a goal of down-listing the species to threatened by 2035. One criterion for down-listing the species requires the AWBP to maintain a population of at least 1000 individuals [3]. A second criterion relaxes this requirement to at least 400 individuals in the AWBP if a second self-sustaining flock is established [3]. Population projections indicate the probability of the AWBP reaching 400 individuals by 2040 is greater than 80% [4], however the probability of this population reaching 1000 individuals by 2040 is essentially zero [4], and the AWBP is not likely to reach 1000 individuals until at least the mid-2060s [5]. The species therefore remains endangered and is highly susceptible to stochastic events that could decimate the population.

Disease is cited as one of the factors for listing the whooping crane as endangered [3], however little is known about diseases affecting these birds. A variety of infectious diseases have been reported in captive and reintroduced whooping cranes [6, 7], however similar studies for wild cranes are lacking. To our knowledge, a 1978 publication is the only published report concerning bacterial, viral, or parasitic pathogens affecting the AWBP whooping cranes, and that study analyzed a single fecal sample each from 19 individuals. In that publication, nearly one third of cranes sampled were shedding coccidia [8].

Coccidia are obligate intracellular protozoan parasites in the phylum Apicomplexa. Coccidian parasites in the genus *Eimeria* infect a wide range of vertebrate and invertebrate hosts [9]. *Eimeria* have a direct fecal-oral life cycle. Noninfective oocysts are passed in the feces and undergo sporulation in the environment to become infective. Oocysts are hardy and can survive a wide range of environmental conditions. The sporulated oocyst (sporocyst) is ingested in food or water and undergoes asexual and sexual reproduction in host epithelial cells. Oocysts are the product of sexual reproduction and are excreted in the feces, and detecting oocysts within voided fecal samples is the most common method of diagnosing coccidian infection of a host [9]. The majority of *Eimeria* species infect intestinal epithelial cells, and the remainder infect renal epithelial cells, with few exceptions [10]. The *Eimeria* species infecting cranes (*E*. *gruis* and *E*. *reichenowi*) are two such exceptions. Unlike the common poultry-associated *Eimeria* species that cause localized infections and variable enteric disease, *E*. *gruis* and *E*. *reichenowi* can spread systemically in cranes, causing disseminated visceral coccidiosis (DVC) [11, 12]. Clinical signs of DVC depend on the tissues affected and the severity of infection, and can include enteritis, hepatitis, bronchopneumonia, myocarditis, and splenitis. Oocysts develop in the intestine or respiratory tract and are shed in the feces [13]. Chronic infections are characterized by granulomas disseminated throughout many organs [11]. DVC is an important cause of crane chick mortality in captivity [12, 14–16], and has also been described in captive adult cranes [17]. In one study, experimentally infected sandhill crane (*Grus canadensis*) chicks all developed granulomas, and 23.8% of wild sandhill cranes had granulomas at necropsy [18]. A separate study found 84% of wild sandhill cranes that had granulomas were also shedding oocysts in the feces [19]. Wild whooping crane chicks are associated with high mortality (27%- 68%) during the first 20 days after hatching [20–22], and the role of DVC as a cause of wild chick mortality is poorly understood [11]. Additionally, infection with these *Eimeria* species may make surviving birds more susceptible to other disease or predation [12].

*Eimeria* species have been described in at least eight species of cranes [11] worldwide, and probably infect all crane species, however, only *Eimeria gruis* and *Eimeria reichenowi* are diagnosed commonly [14]. *E*. *gruis* and *E*. *reichenowi* have been described in wild and captive whooping, sandhill, white-naped (*Grus vipio*), and red-crowned cranes (*Grus japonensis*), and additionally in captive demoiselle (*Anthropoides virgo*), sarus (*Antigone antigone*), and Eurasian cranes (*Grus grus*) [11, 16]. Phylogenetically, the *E*. *gruis* and *E*. *reichenowi* that were isolated from hooded, white-naped, and red-crowned cranes cluster in a clade separate from the *Eimeria* species infecting other birds and mammals [23, 24], but the genetics of *Eimeria* infecting whooping cranes has not previously been explored. Here, our objectives were to (*i*) determine the prevalence and phenology of coccidia shedding in the wintering AWBP population of whooping cranes; (*ii*) compare microscopic and molecular detections of coccidia species; and (*iii*) determine the phylogenetic relationships among the *E*. *gruis* and *E*. *reichenowi* isolated from whooping cranes to those from other crane species, and other *Eimeria* species.

## Methods

### Ethics Statement

Fecal samples were collected on the Aransas National Wildlife Refuge under Special Use Permit #21531-13-003 and #21530-14-03-DI. Because this study used only voided fecal samples, and no manipulation of live animals, the Texas A&M University Institutional Animal Care and Use Committee issued an exemption for our study.

### Fecal sample collection

**Fig 1:**
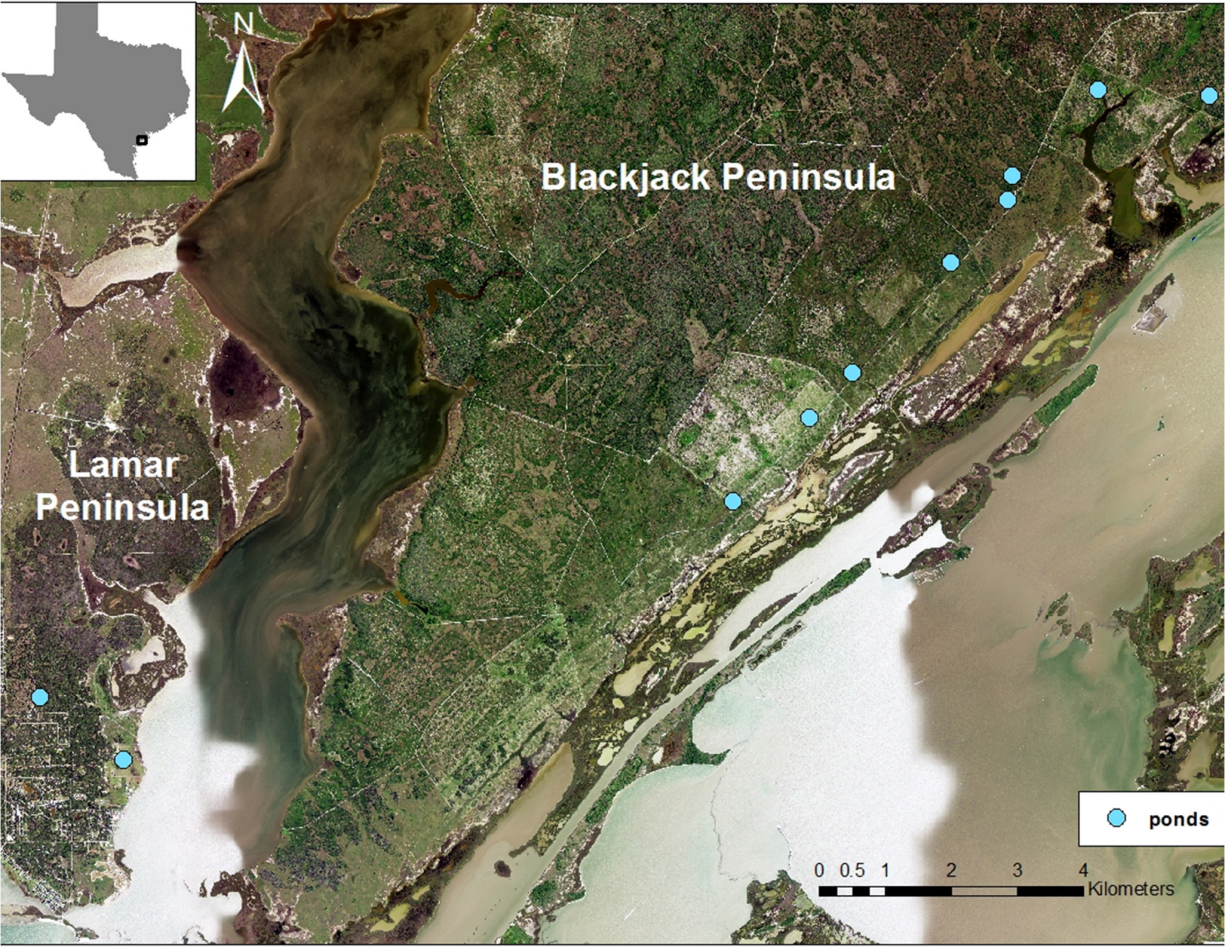
Pond Sites on the Blackjack and Lamar Peninsulas. The Aransas National Wildlife Refuge is located along the Texas Gulf Coast and encompasses the Blackjack Peninsula and Matagorda Island. Eight pond sites in this study were located on the Blackjack Peninsula. The two pond sites on the Lamar Peninsula were included during 2012–2013. The map image was created by the USDA National Agriculture Imagery Program (NAIP) and downloaded as a GIS file, and the figure was produced using ArcMAP 10 (Esri, Redlands, CA).

**Fig 2:**
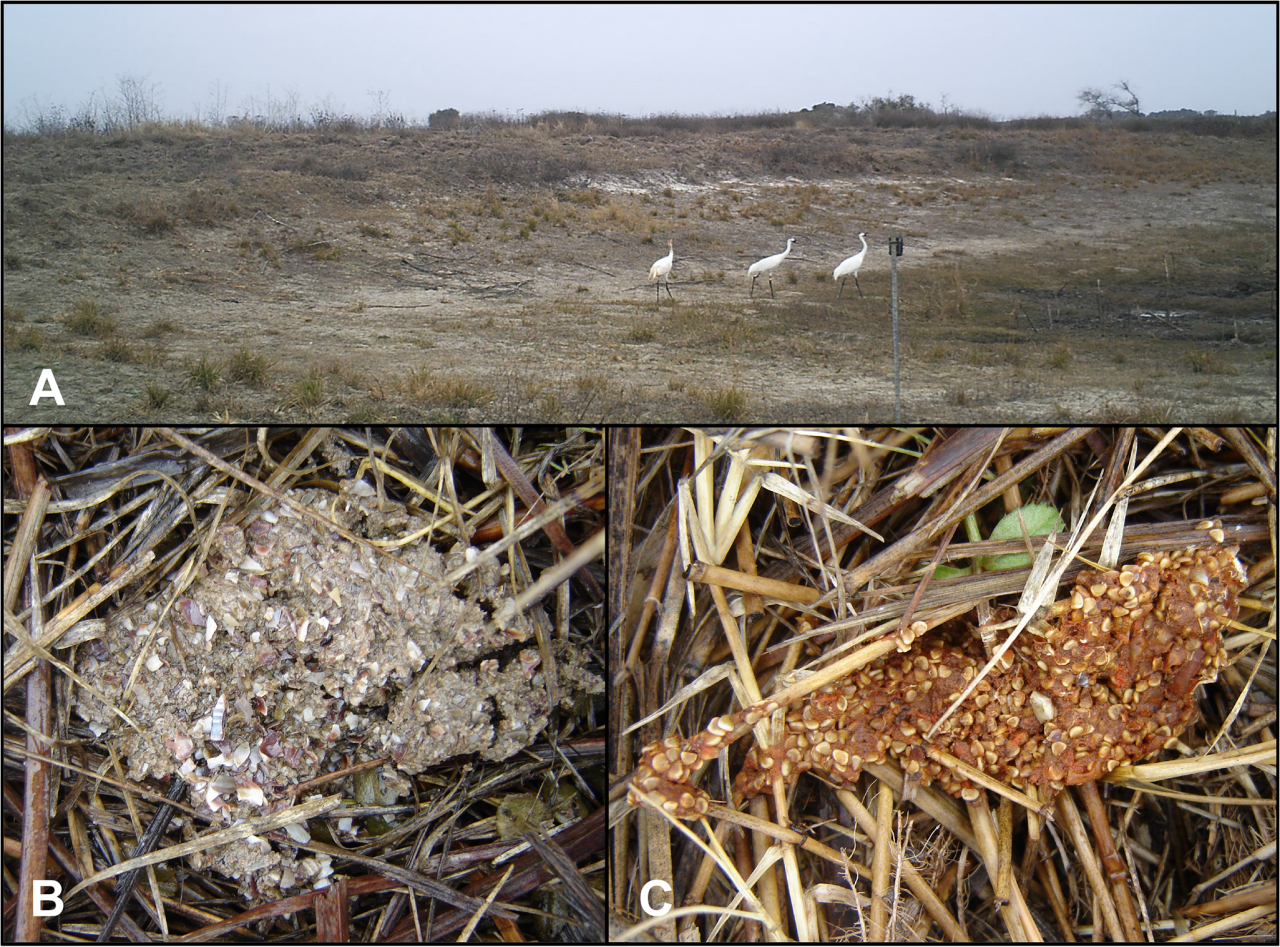
Whooping Crane Scat Collection. Game camera photo of two adult whooping cranes and one juvenile (far left) at a freshwater pond on Aransas National Wildlife Refuge (A). Scat produced by a whooping crane feeding primarily on blue crab and other invertebrates (B) and wolfberry (C). Cranes fed predominantly on wolfberry when the berries were abundant (Nov—Dec), then switched to feed predominantly on crab and other invertebrates. Scat was collected after the cranes naturally left the pond.

Whooping crane fecal samples were collected every three weeks during two winter seasons, Nov 2012-March 2013 and Nov 2013-April 2014, at the Aransas National Wildlife Refuge in Aransas, Refugio, and Calhoun counties on the Texas Gulf Coast (28.313449,-96.804022)(Fig 1). Because invasive sampling for health surveillance of these endangered birds is not desired due to their conservation status, our sampling approach is based on analyses of voided fecal samples. Fecal samples were collected from a series of ten artificial freshwater ponds on the refuge. Whooping cranes utilize freshwater sources for drinking when salinity levels in the marsh are high [25], as during the ongoing drought conditions in Texas which persisted through the study period. Although cranes are distributed across the refuge on territories, we prioritized fecal searching and collection at the ponds for the following reasons: (i) multiple family groups use the same ponds such that many individuals may be sampled from the same focal area; (ii) ponds are accessible from land and did not require boats to access; and (iii) disturbance to the birds due to the presence of our research team was limited because all ponds were located along the main service road and birds are habituated to occasional traffic along this road. Additionally, aggregation of cranes at the ponds may facilitate parasite transmission, resulting in higher prevalence of infection. We deployed infrared game cameras (Trophy Cam HD, Bushnell, Overland Park, KS) to determine patterns of whooping crane use of the ponds, and planned our collection excursions to occur after cranes departed. We collected fresh (estimated to be <24 hrs old) feces from 8 pond sites on the Blackjack peninsula during the study, and from an additional 2 pond sites on the Lamar peninsula during the 2012–2013 season (Fig 1). The two sites on the Lamar peninsula yielded very few samples and were eliminated from the sampling sites in the 2013–2014 season. We searched each pond site for fresh feces twice during each two-day collection trip. Feces were collected into Whirl-pak bags, after which air was removed manually, and samples were stored on ice for transportation and storage at 4^°^C in the lab. Feces were selected for collection when they met the appearance of whooping crane scat (Fig 2) based on food contents (blue crab and wolfberry [26, 27]) and in combination with evidence of recent whooping crane presence at the pond (tracks, game camera pictures). Sandhill cranes cohabitate with whooping cranes in the study area, and we also collected several sandhill crane fecal samples for comparison.

### Host species confirmation

To confirm host species, the *Grus* genus-wide primers Grus16SF and Grus16SR [28] were used to amplify a 470-bp fragment of the mitochondrial 16S rRNA gene for a systematic random sample of 10% of fecal samples that we suspected were from whooping cranes based on field observations. Additionally, for proof-of-principle, we also analyzed eight fecal samples that we suspected were from sandhill cranes. PCR was performed in 15 μl reactions consisting of 1X FailSafe PCR Premix A, 0.15 μl FailSafe Enzyme, 0.25 μM each primer, 0.1 μg/μl BSA, and 1 μl fecal DNA. Cycling parameters were as described previously [28]. Positive samples were purified using ExoSAP-IT (Affymetrix, Santa Clara, CA) according to manufacturer’s instructions. Purified samples were submitted for bi-directional sequencing to Eton Bioscience Inc. (San Diego, CA). Sequences were compared to known crane sequences using the BLAST tool in GenBank, and a representative sequence of a whooping and a sandhill crane were deposited in GenBank (Accession #KP966312 and KP966313).

### Fecal flotation

All samples were subjected to fecal flotation within 5 days of collection following standard veterinary protocol [29]; our own trials suggested the ability to detect coccodia microscopically and molecularly was not altered within this period (unpublished data). Briefly, one gram of feces was suspended in 10 ml zinc sulfate solution (s.g. 1.18), strained through a double layer of gauze, and transferred to a 15 ml centrifuge tube. Zinc sulfate solution was added to a final volume of 15 ml and a coverslip was placed over the top of the tube. Samples were centrifuged in a swinging-bucket centrifuge at 2000xg for 5 minutes. The coverslip was immediately placed on a slide and examined for the presence of coccidia oocysts using a compound light microscope. The entire coverslip was examined at 125X magnification, and suspected oocysts were further examined and measured at 500X magnification. Oocyst shape (pyriform or round/oval), presence and number of sporocysts in the oocysts, burden of infection, and single vs. mixed species infection were noted. Burden of infection was defined qualitatively as low (<2 oocysts per high power field), medium (2–10 oocysts/hpf), or high (>10 oocysts/hpf).

### Molecular detection of coccidia

All samples (regardless of fecal flotation result) were subjected to a second fecal flotation to generate a template with concentrated oocysts for DNA extraction. We modified the flotation procedure described above by spinning tubes without a coverslip. Immediately after centrifugation, 100 μl of liquid at the surface (which would contain concentrated oocysts in positive samples) was transferred from the surface of the tube into a microcentrifuge tube. During the 2012–2013 season, the resulting samples were immediately stored at -20°C until DNA extraction. During the 2013–2014 season, the resulting samples were washed twice with 300 μl water to remove residual zinc sulfate and then stored at -20°C until DNA extraction. The DNA was extracted using the QIAmp DNA Stool Mini Kit (Qiagen, Valencia, CA) following manufacturer’s instruction during the 2012–2013 season. During the 2013–2014 season, DNA was extracted using the E.Z.N.A. Stool DNA Extraction Kit (Omega Biotek, Norcross, GA), and samples were processed in a cell disruptor (Mini-beadbeater 96, BioSpec Products, Inc., Bartlesville, OK) for 90 seconds to break open the oocysts, then incubated at 55°C overnight. We then proceeded with DNA extraction following the manufacturer’s instructions. All samples were eluted in two rounds of 25μl (50 μl total) into the same tube.

Coccidia were detected using PCR to amplify a portion of the internal transcribed spacer regions (ITS) using two previously published assays. A 466-bp region of the first internal transcribed spacer (16S–5.8S rRNA region) was amplified using the primers BSEF and BSER [30] at a concentration of 0.5 μM in a 15 μl reaction. Remaining reaction components consisted of 1X FailSafe PCR Premix B (Epicentre, Madison, WI), 0.15 μl FailSafe Enzyme, 0.1 μg/μl BSA, and 1 μl of sample template. Cycling parameters were as described by [31], except annealing temperature was changed to 55°C because this temperature was determined to be optimal after our pilot trials. Alternatively, a nested reaction was used to amplify a 400-bp region of the second internal transcribed spacer (5.8S–28S rRNA region). The initial PCR used the primers EITSF2 and EITSR2 [28] at a concentration of 0.25 μM in a 15 μl reaction. Remaining reaction components consisted of 1X FailSafe PCR Premix A, 0.15 μl FailSafe Enzyme, 0.1 μg/μl BSA, and 1 μl sample template. The second PCR used the primers WW2 and WW4r [32] at 0.25 μM in a 15 μl reaction. The first PCR product was diluted 1:50 and 1 μl of the diluted product was used in the second PCR. All other reaction components were identical to the first PCR. Cycling parameters were run as previously described [28].

We used an independent PCR for a different *Eimeria* gene on a random subset of positive samples and negative samples for confirmatory purposes. The primers 1FE and 4RB [33] were used to amplify a 358-bp region of the 18S rRNA gene at a concentration of 1 μM in a 15 μl reaction. Remaining reaction components consisted of 1X FailSafe PCR Premix E, 0.15 μl FailSafe Enzyme, and 1.5 μl fecal DNA. Cycling parameters were run as previously described [23].

To complement the morphological differences we noted between crane-associated *Eimeria* species, we confirmed the identity of coccidia species using DNA sequencing for all three amplified regions (ITS-1, ITS-2, and 18S rRNA). All positive sequences were purified and sequenced as described above. Forward and reverse sequences were aligned and a consensus sequence was determined using Clustal W within Mega 6.0 [34]. Sequences were compared to known *Eimeria* sequences using the BLAST tool in GenBank. Consensus sequences were then aligned along with publicly available *Eimeria* species sequences and analyzed in Mega 6.0 using a neighbor-joining tree using the bootstrap method with 1000 replicates. Samples with poor quality sequences or multiple peaks were excluded from phylogenetic analysis. All sequences produced during this project and utilized in the phylogenetic analysis were deposited in GenBank (Accession #KP966299—KP966311).

### Statistical Analysis

Statistical analysis was performed using SAS software, version 9.4 (Cary, NC). Proportion of samples positive and confidence intervals were calculated accounting for clustering at the pond level. The chi-squared test was used to compare proportion of positive samples between ponds and between years. Fisher’s exact test was used to compare proportion of positive samples between dates of collection within each year due to small samples sizes during 2013–2014. Proportion of samples positive based on microscopy was compared to that based on PCR using the chi-squared test.

## Results

### Sample collection and host confirmation

We collected a total of 339 fecal samples, with 227 collected during 2012–2013 and 112 collected during 2013–2014. Of these, 11 were suspected to come from sandhill cranes, whereas the remainder was attributed to whooping cranes based on visual characteristics. A total of 79 samples, including 9 that were suspected to come from sandhill cranes, were subjected to a molecular confirmation of host species. All 9 that were suspected to come from sandhill cranes based on appearance were confirmed to contain sandhill crane DNA based on DNA sequence analysis; sandhill crane samples were excluded from further analysis. Of the remaining 70 samples, 37 were confirmed to contain whooping crane DNA based on sequence analysis and 5 sequences were poor quality and could not be matched to species. The remainder of samples did not amplify using PCR, but nonetheless are included in the analysis as originating from whooping crane due to field identification and presence of whooping cranes immediately preceding collection based on camera trap and observational data. The lack of amplification could be attributed to a lack of host DNA in the floated fraction of fecal material that was subjected to DNA extraction, or degradation of host DNA in the feces while in the field.

### Microscopic examination

**Fig 3:**
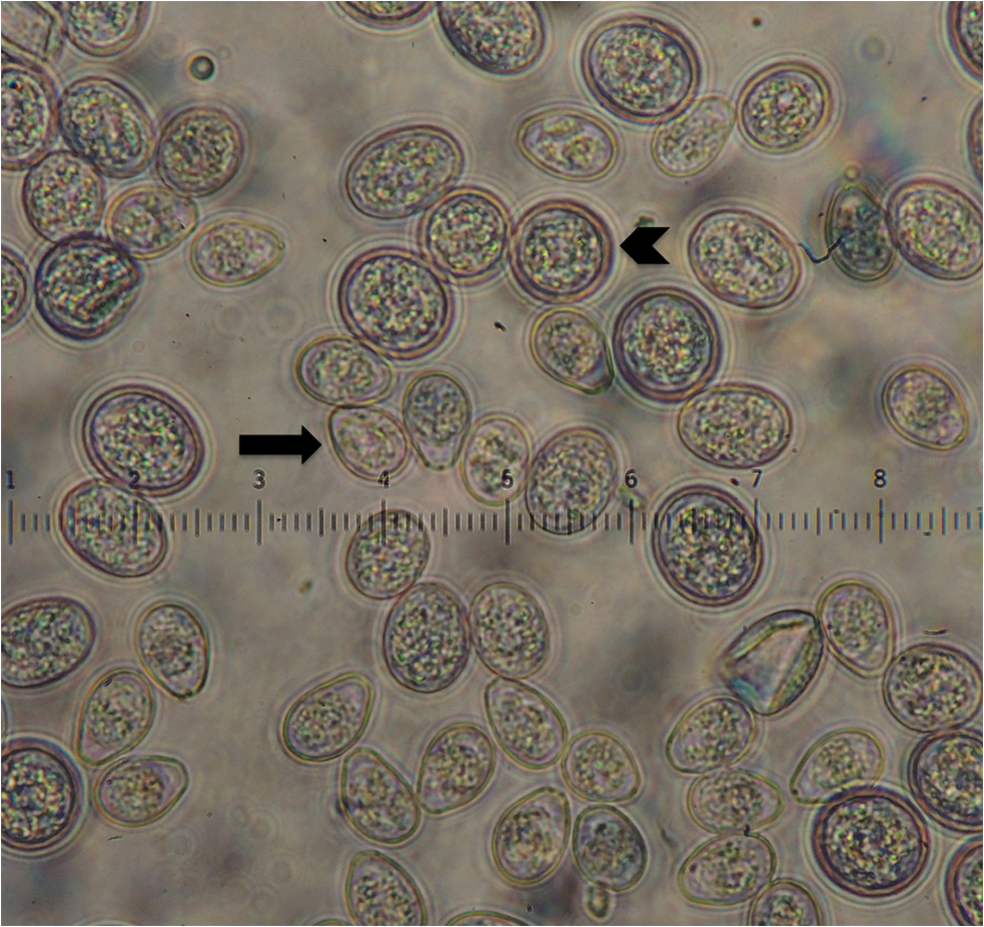
Coccidia Observed during Fecal Flotation. Fecal flotation under 500X magnification showing a mixed infection with two species of *Eimeria*. The smaller, pear-shaped oocysts are consistent with *Eimeria gruis* (arrow) and the larger, round to oval oocysts are consistent with *Eimeria reichenowi* (arrowhead).

We identified two types of oocysts based on morphology (Fig 3). The first type was pyriform and measured 18 μm x 12 μm (range 16–20 μm x 10–14 μm), and matched size descriptions of *Eimeria gruis* [35, 36]. The second type was round to oval and measured 20 μm x 16 μm (range 12–22 μm x 12–20 μm), and matched size descriptions of *Eimeria reichenowi* [35, 36].

In total, 87 of 328 (26.5%; 95% CI: 20.3%–32.8%) samples were positive for *Eimeria* on microscopy. The majority of samples (63.2%, n = 55) had a low burden of infection, 20 (23%) had a medium burden of infection, and 12 (13.8%) had a high burden of infection. The burden of infection did not differ significantly across the study (p = 0.22, χ^2^ = 4.45, df = 3). Fifty-seven samples (65.5% of positive samples) were single infections with *E*. *gruis*, 17 (19.5%) were single infections with *E*. *reichenowi*, and 12 (13.8%) were mixed infections with both *Eimeria* species. Data for the two species were combined for further analysis due to low sample numbers for *E*. *reichenowi*. There was no significant difference in overall proportion of positive samples between 2012–2013 and 2013–2014 (29.3% and 20.8%, respectively; p = 0.08, χ^2^ = 3.17, df = 1). The proportion of *Eimeria* positive samples varied significantly across the season during 2012–2013 (Fisher’s exact test, p<0.001), but not during 2013–2014 (Fisher’s exact test, p = 0.361). Across the November-April winter collection season, prevalence peaked in December and again in April during 2012–2013, whereas there was a single peak in January during 2013–2014 (Table 1). The proportion of *Eimeria* positive samples did not vary significantly among ponds (p = 0.43, χ^2^ = 9.10, df = 9).

**Table 1:** Phenology of *Eimeria* shedding in winter based on microscopy in whooping crane feces.

	2012–2013		2013–2014	
	Positive Samples (%)	Total Samples	Positive Samples (%)	Total Samples
November	8 (2.0)	25	5 (18.5)	27
December	27 (58.7)	46	5(25.0)	20
January	11 (23.4)	47	2 (50.0)	4
February	8 (12.7)	63	6 (18.2)	33
March	4 (14.8)	27	3 (33.3)	9
April	7 (50.0)	14	1 (7.7)	13

### Molecular examination

Samples that were very dry or very small (n = 66) were excluded from molecular analysis. In total, 43 of 262 (16.4%; 95% CI: 10.5%–22.4%) samples were positive for *Eimeria* using PCR for either ITS-1 or ITS-2 regions. The proportion of samples that tested positive during the 2012–2013 season was 8.9% (95% CI: 1.7%–16.1%; n = 157), and was significantly less than during the 2013–2014 season (27.6%; 95% CI: 13.2%–42.0%; n = 105; p<0.0001, χ^2^ = 14.66, df = 1). The large difference between years was attributed to the improved sample preparation and DNA extraction protocol we used in the 2013–2014 season.

A total of 41 samples was subjected to PCR for *Eimeria* 18S rRNA for an independent assessment. Of the 38 that were positive based on PCR for either ITS region of *Eimeria*, 28 (73.7%) were also positive in the 18S rRNA PCR. Of the 3 that were negative based on PCR for the ITS region, 2 (66.7%) were also negative in the 18S rRNA PCR.

The proportion of samples that tested positive as determined by PCR for ITS-1 or ITS-2 was significantly lower than that which was determined by microscopy during 2012–2013 (p<0.0001, χ^2^ = 22.13, df = 1), but during 2013–2014, the proportion of positive samples as determined by PCR for ITS-2 was significantly greater than that determined by microscopy (p<0.0001, χ^2^ = 46.23, df = 1). During 2013–2014, PCR had a sensitivity of 86.4% and a specificity of 88.0% compared to microscopy for detection of coccidia. Of the 105 samples collected during 2013–2014, 19 samples were positive and 73 samples were negative based on both microscopy and PCR, whereas 3 samples were positive on microscopy but negative on PCR, and 10 samples were negative on microscopy but positive on PCR.

### Phylogenetic analysis

**Fig 4:**
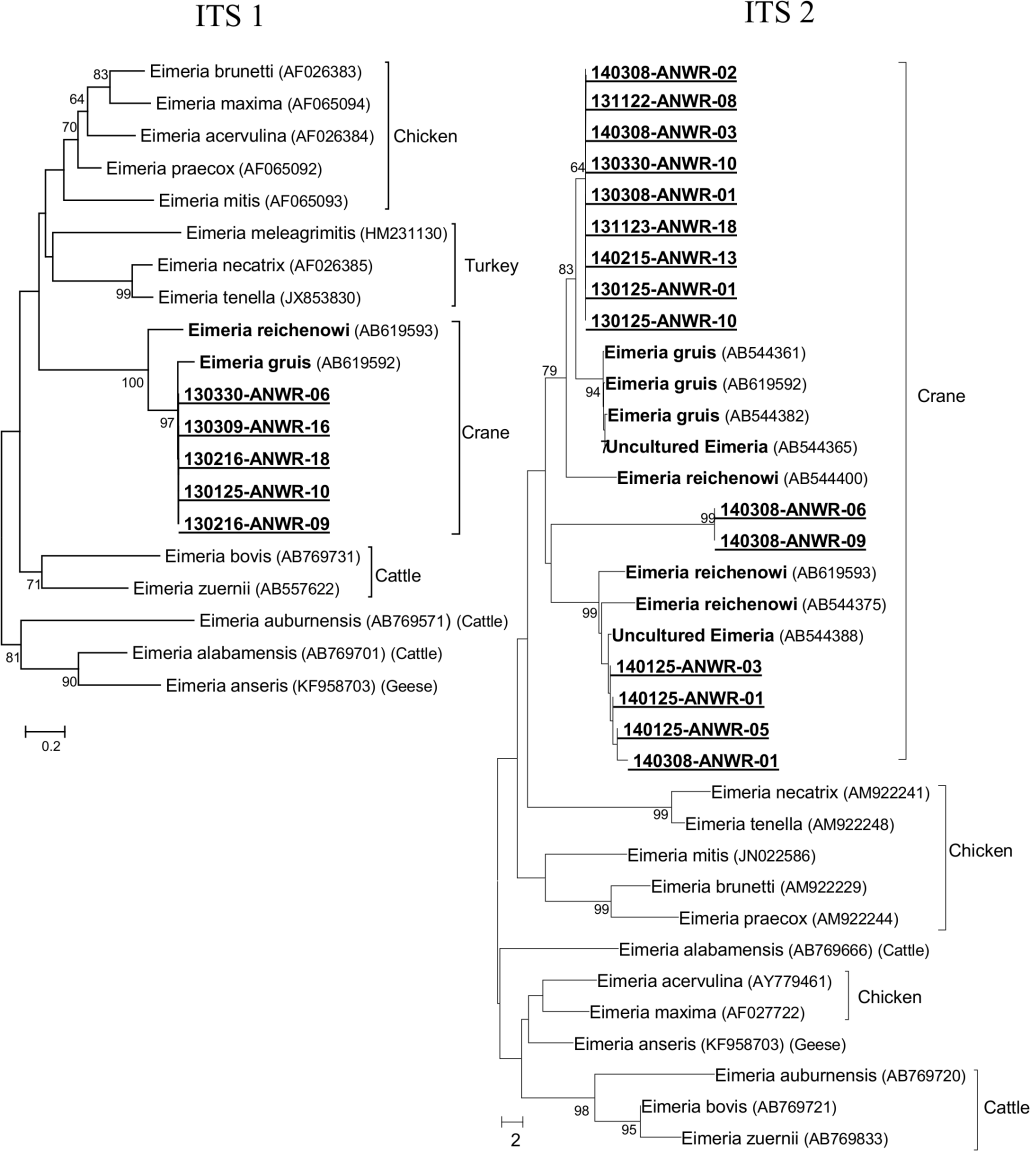
Phylogenetic Tree using *Eimeria* ITS-1 and ITS-2 Sequences. Phylogenetic trees using the neighbor-joining method on ITS-1 (466 bp) or ITS-2 (400 bp) sequences from *Eimeria* species. Bootstrap values are based on 1000 replicates and shown where greater than 60. Bold species indicate isolates from cranes, and underlined species indicate sequences generated in this study. The GenBank accession number of each isolate is shown in parentheses, and the known vertebrate host is also shown.

We obtained forward and reverse DNA sequences from either the ITS-1 or ITS-2 regions from 21 samples to include in the phylogenetic analysis. The five samples for which we determined ITS-1 sequences were identical to each other and matched closely with a previously published *E*. *gruis* sequence from a hooded crane in Japan [28], and the crane-associated clade is more closely related to poultry *Eimeria* species than to cattle *Eimeria* species (Fig 4). The 15 ITS-2 sequences produced similar results, with the crane *Eimeria* species forming a separate clade that was more closely related to poultry *Eimeria* species than to cattle *Eimeria*. However, our ITS-2 sequences showed three distinct lineages. One lineage, comprised of nine nearly identical sequences, grouped with previously published *E*. *gruis* sequences. Another lineage was comprised of four sequences and grouped with previously published *E*. *reichenowi* sequences. The third lineage, comprised of two identical sequences, formed a unique group within the crane *Eimeria* clade.

**Fig 5:**
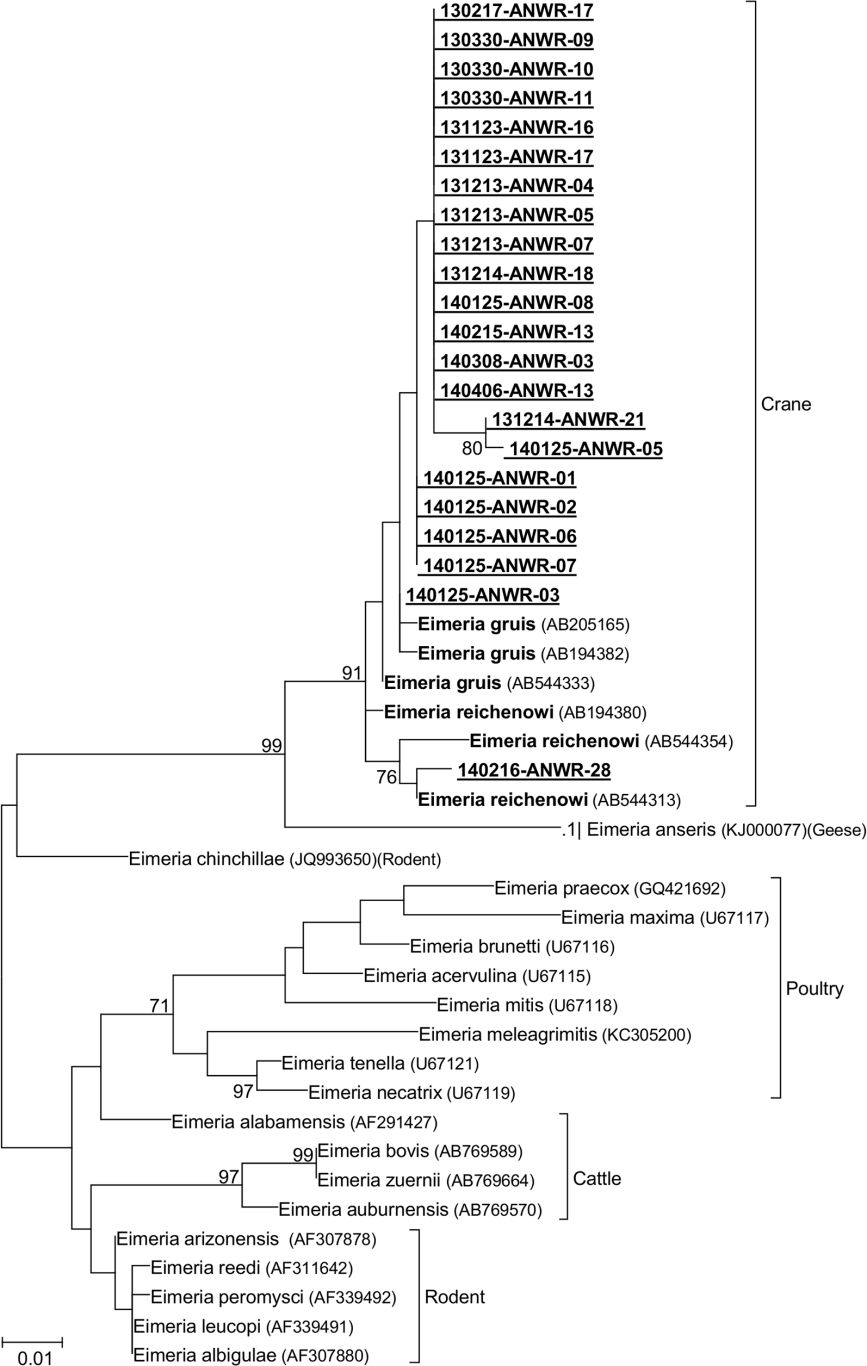
Phylogenetic Tree using *Eimeria* 18S rRNA Sequences. Phylogenetic tree using the neighbor-joining method on 18S rRNA sequences (358 bp) from *Eimeria* species. Bootstrap values are based on 1000 replicates and shown where greater than 60. Bold species indicate isolates from cranes, and underlined species indicate sequences generated in this study. The GenBank accession number of each isolate is shown in parentheses, and the known vertebrate host is also shown.

We obtained forward and reverse DNA sequences from the 18S rRNA gene for 28 samples. Upon manual examination of the chromatograph traces, six samples had double nucleotide peaks at two polymorphic sites within the alignment that were among those that differentiated *E*. *gruis* and *E*. *reichenowi* and were excluded from phylogenetic analysis. All analyzed sequences were within the clades that contained the previously published *E*. *gruis* and *E*. *reichenowi* sequences. The crane *Eimeria* species formed a clade with *E*. *anseris* from domestic geese that was separate from all other *Eimeria* species investigated. Using 18S rRNA sequences, *Eimeria* species from poultry, cattle, and rodents are more closely related to each other than to crane *Eimeria* (Fig 5). Two of the three *E*. *reichenowi* published sequences (from a crane in Japan) and the sequence we generated from a whooping crane formed a unique clade. The third *E*. *reichenowi* published sequence formed a separate branch in the crane *Eimeria* clade. Among samples (n = 10) for which we generated both ITS and 18S rRNA sequences, the *Eimeria* species assignment was congruent based on analysis of both loci for all but two samples (140125-ANWR-01; 140125-ANWR-03), which grouped with *E*. *reichenowi* at the ITS locus and grouped with *E*. *gruis* at the 18S rRNA locus. Microscopic assessment of the oocysts in these two samples revealed both round and pear-shaped oocysts, indicative of mixed species infections.

Overall, DNA sequence analysis supported morphologic analysis for species-level identification. Samples containing the pear-shaped oocysts on microscopy aligned with *E*. *gruis* on the DNA sequence analysis. Several samples that contained both types of oocysts, but had many more pear-shaped than round oocysts, also aligned with *E*. *gruis* on DNA sequence analysis. One of the samples containing only the round oocysts produced a good quality sequence and aligned with *E*. *reichenowi* on the DNA sequence analysis.

## Discussion

We document that nearly one-third of fecal samples collected from the only wild migratory population of whooping cranes on their wintering grounds harbor *Eimeria* species coccidian parasites, based on visualization of oocysts and PCR analysis of voided fecal samples. These data underscore the importance of understanding how coccidian parasites may impact population health. Our findings are similar to those reported in the only previous published assessment of coccidia in this population of whooping cranes (31.5%)[8]. Although these two datasets suggest that coccidia infection may have remained stable while this population increased in size over the past 35 years, the study from the 1970s was based on a single fecal sample from only 19 individuals. Although the exact number of individual cranes represented in our analysis is not known, the ponds from which we collected samples are utilized by birds of multiple family groups. During December and January 2012–2013, approximately 17 individuals were documented in the marshes adjacent to our study sites, increasing to 45 individuals in late February (Elizabeth Smith, personal communication); we therefore expect our study samples represent a subset of this number of birds. In contrast to the AWBP whooping cranes, only 13% (n = 54) of reintroduced whooping cranes in Florida were found to be shedding coccidia [37]. The reintroduced cranes had access to feed containing a coccidiostat, which likely explains the lower prevalence of *Eimeria* among this population [37]. In other avian host species, oocyst shedding can vary with the time since infection and the time of day when the feces is voided [38, 39]. Numerous studies of *Eimeria* and *Isospora* species in other avian hosts have shown that oocyst shedding is lowest in the morning and increases through the day [40–42]. Although we collected samples in the mid- to late-afternoon each day, the samples were voided by cranes throughout the day. If *Eimeria* in cranes follow the same diurnal shedding pattern, oocysts may not be present in fecal samples deposited in the morning even if the crane is infected. Furthermore, infected birds may not shed oocysts across the full time frame of infection. Novilla et al. [43] found oocysts in fecal samples from three of four captive sandhill cranes with DVC, and a study of hunter-harvested wild sandhill cranes found oocysts in fecal samples of 84% (n = 64) of cranes with DVC [19]. Accordingly, if we assume the fecal samples we studied are representative of the crane population on the refuge, our results suggest the true coccidia infection prevalence in the whooping cranes is likely higher than the results of our fecal analysis indicate.

Through a longitudinal assessment, we found that prevalence of coccidia shedding varied across the season, but the variation was not consistent across the two years of the study. During the first year, prevalence peaked in December and again in April, however there was only a single nonsignificant peak in January during the second year. The lack of a significant trend in the proportion of positive fecal samples during 2013–2014 may be due to small sample sizes during January and March. Our results suggest that birds arrive infected and maintain a low level of shedding throughout the winter season. Interestingly, previous studies in wild red-crowned cranes (*Grus japonensis*) in Japan found a similar level of *Eimeria* infection in fecal samples (26%) and a higher percentage of infection in samples collected in December compared to January through April [44]. The authors of that study suggest two possible explanations for the decrease in *Eimeria* infection over the winter: 1) temperatures are too cold for sporulation to occur, therefore new infections do not occur; and 2) coccidiosis is a self-limiting disease, and recovered cranes no longer shed oocysts [44]. We suggest other factors must be involved in the phenology of oocyst shedding in our study, since winter temperatures along the Texas Gulf Coast remain mild enough for sporulation to occur [45], and *E*. *gruis* and *E*. *reichenowi* spread systemically, unlike other *Eimeria* species. Viable schizonts have been seen in granulomas in multiple tissues, which potentially prolong the infection [18]. Hartman et al. [46] documented temporal peaks in shedding of *E*. *gruis* and *E*. *reichenowi* in fecal samples collected from communally roosting sandhill cranes in Wisconsin during the summer. Temporal shedding and communal roosting likely increase transmission, and communal roosting is common among whooping cranes at the ANWR. We are currently investigating the degree to which physiological stress may contribute to *Eimeria* shedding.

The discrepancy we observed in prevalence estimates based on microscopic vs. molecular examination during 2012–2013, in which significantly more matched samples were positive using microscopy, is likely attributed to an inefficient DNA extraction protocol used in the 2012–2013 season. Specifically, we did not include a mechanical breaking step, as we were following the protocol of Honma et al. [28] and assumed there may be enough free DNA released from opened or degraded oocysts. We refined our extraction method during 2013–2014 to include a mechanical breaking step in addition to rinsing oocysts to remove excess flotation solution since high salt concentrations are detrimental to PCR reactions. With these modifications, we found that the proportion of positive samples was greater than we detected in the previous field season. Furthermore, more samples were determined to be positive based on PCR than on microscopy. Honma et al. [28] concluded that PCR is less sensitive than microscopy, however we showed that with proper sample preparation, PCR can detect more positive samples than microscopy, and may be used as a conservation tool to monitor the prevalence of *Eimeria* in the whooping crane population.

The ITS regions can be used to determine the species of *Eimeria*, however there are multiple copies of these regions in the *Eimeria* genome, and sequence length can vary within a single oocyst, limiting the utility of these regions for investigating phylogeny [9, 47]. The 18S rRNA gene is more conserved, making it more suitable as a marker for both species identification and phylogenetic analysis. Previous studies have shown that the *E*. *gruis* and *E*. *reichenowi* that infect cranes in Japan are phylogenetically distinct from other *Eimeria* species [23, 24], and our results show this is true for the *E*. *gruis* and *E*. *reichenowi* that infect cranes in North America. Furthermore, many 18S rRNA sequences from our study are identical to each other, but distinct from previously published *E*. *gruis* and *E*. *reichenowi* sequences, suggesting there may be different lineages of these parasites infecting cranes in North America and in Japan. Although recent studies on *Eimeria* species that infect poultry indicate that the 18S rRNA gene is not suitable by itself for identification and phylogenetic analysis at the species level, and propose using the cytochrome *c* oxidase subunit I (COI) gene instead [48, 49], we elected to use the ITS regions and 18S gene in this study because they have been characterized for crane-associated *Eimeria* species, whereas COI has not.

We found that searching for freshly-voided fecal samples around freshwater ponds at ANWR is an efficient means of collecting a large number of fecal samples. One drawback to this method, however, is that it is difficult to match samples to individual birds at the time of collection because these ponds are used communally by a large number of birds. An alternative means of collection is to monitor family groups on territories in the marshes and search for feces after the birds have vacated an area. This method is time-consuming and yields low numbers of samples [50], but may be necessary to represent a large number of individual birds during wet years when pond use is diminished.

We have detected a high and persistent prevalence of coccidian parasites in whooping cranes, and the degree to which parasites regulate the whooping crane population remains unknown. Previous studies provide a framework for understanding the potential for parasites to regulate wild vertebrate host populations. For example, long-term experimental reductions in the burden of a parasitic nematode resulted in increased fecundity and a prevention of population crashes of free-living red grouse (*Lagopus lagopus scoticus*) in England [51]. A meta-analysis investigating the effect of parasites on wild vertebrates revealed a significant negative effect of parasites at the population-level which resulted from reduced clutch size, hatching success, young produced, and survival [52]. Current evidence indicates coccidia infecting cranes frequently spread systemically to cause DVC [11, 18, 19], although mortality is low in adult birds [37]. Mortality from DVC is likely much higher in chicks, and the disease may exert a population-level effect by reducing survivorship of this life stage. However, the cause of death of chicks is exceedingly difficult to ascertain due to the remote location of the breeding grounds in Wood Buffalo National Park in northern Canada. The prevalence of coccidian parasites within whooping crane fecal samples at the key refuge used for overwintering of the species underscores the importance for considering DVC as a disease that may be regulating the population growth of this species. Understanding the times and locations important in *Eimeria* transmission will aid conservation efforts and inform management decisions aimed at the recovery of the AWBP whooping cranes.
